# Editorial: Insights in geriatric medicine: 2021

**DOI:** 10.3389/fmed.2023.1347154

**Published:** 2024-01-09

**Authors:** Tzvi Dwolatzky

**Affiliations:** ^1^Geriatric Unit, Rambam Health Care Campus, Haifa, Israel; ^2^Ruth and Bruce Rappaport Faculty of Medicine, Technion – Israel Institute of Technology, Haifa, Israel

**Keywords:** geriatrics, frailty, COVID-19, delirium, functional status

The COVID-19 pandemic caught the world by surprise. The initial response was largely one of uncertainty, instability, even panic (1). As countries began to gain insight into the behavior of a highly contagious virus with serious health consequences on the individual and on health care services, responses became more organized and focused. On this background of the COVID-19 pandemic, Frontiers in Medicine took the lead in providing clinicians and scientists an opportunity to share their innovative research involving older people in a special Research Topic entitled *Insights in Geriatric Medicine: 2021*. The impact of the pandemic on this Research Topic was clear, with the submission of a number of studies relating specifically to the older population and COVID-19. However, it was encouraging to see the submission of an array of scientific research focusing on many aspects of the health of older people. The resulting Research Topic is interesting and innovative.

Focusing on COVID-19, Kyriazis et al. describe the consequences of prolonged lockdown and social distancing on older people in Cyprus. The authors found an increased risk of death from causes other than COVID-19 in older people. The authors call for a clear policy based on a comprehensive multifaceted approach, including medical, social, physical and psychological elements.

An interesting and timely meta-analysis that estimated the prevalence of insomnia symptoms in older Chinese adults during the COVID-19 pandemic evaluated nine studies with a total of 27,207 older Chinese adults. Zhang et al. found that almost one-quarter of those studied reported moderate insomnia symptoms, while 11.1% rated symptoms as severe. Not surprisingly, prevalence rates of insomnia symptoms were significantly higher for those living in the COVID-19 epicenter. The authors correctly highlight the need for supportive mental health services, with a specific focus on the assessment of insomnia symptoms.

Long COVID is increasingly recognized as a true entity that should be characterized and studied further. The study reported by Damanti et al. explored the prevalence of Long COVID-19 symptoms following discharge from admission for acute hospital care. The authors found that compared to robust patients, those who were frail were more likely to be malnourished and had a higher risk of sarcopenia and poor physical performance following discharge from hospital.

Moving away from COVID-19, the collection of manuscripts in this Research Topic also focus on other important areas relating to Geriatric care. One such condition is acute acalculous cholecystitis. A delay in the diagnosis of this condition may result in serious life-threatening complications, such as gangrene or perforation of the gallbladder. Lin et al. retrospectively investigated 374 older bedridden patients with clinical manifestations of acute acalculous cholecystitis. Patients with acute acalculous cholecystitis had a significantly higher incidence of complications, and a longer duration of symptoms and of antibiotic therapy. The authors emphasize the importance of early imaging using recognized diagnostic criteria in older patients with clinical manifestations of acute acalculous cholecystitis.

Acute hospital admission is challenging for older people, who are more prone to delirium and functional decline. The correct approach is clearly one of prevention, since pharmacotherapeutic interventions for delirium are associated with adverse events and have limited efficacy (2, 3). Aomura et al. posed an interesting hypothesis, questioning whether admission to a window-side bed was associated with the development of delirium in older patients admitted to a general ward. Within the limitations of this study, no significant association was found between the position of the hospital bed near a window and the development of delirium.

Recognizing the many factors contributing to increased post-hospitalization mortality, Lattanzio et al. conducted a well-designed multicenter prospective. Dependency in basic activities of daily living and anticholinergic cognitive burden were highly associated with poorer survival at 1 year post discharge, and to a lesser degree those with cognitive impairment had a significant risk of mortality. This study provides an important insight into those risk factors that should be identified and monitored to improve post-hospitalization prognosis.

An important etiological factor leading to acute hospitalization is sepsis. Lang et al. postulate that changes in DNA methylation, which have been shown to be linked to the aging process and to age-related diseases, may play a role in the mechanism and prognosis of sepsis in the older patient. Indeed, they found a significant correlation between 161 CpG methylation sites and poorer outcomes in the older sepsis group. This interesting finding may contribute to improving our understanding as to why older patients are more susceptible to sepsis with poorer outcomes.

The need to undergo major surgery challenges the health care team caring for the older patient. Factors influencing the course of surgery and outcome should be clearly identified. One such factor is polypharmacy, which has been clearly recognized as a geriatric giant with untoward effects on the health and wellbeing of older patients (4). Lertkovit et al. conducted a single-center prospective study to determine the prevalence of polypharmacy in 250 older patients undergoing elective major surgery. While there was no significant association between polypharmacy and postoperative cognitive dysfunction, there was some association between intraoperative therapeutic compounds and postoperative cognitive dysfunction.

The biology of human aging is complex and fascinating. Conte et al. provide an interesting insight onto one of the most interesting aspects of aging, namely the presence of chronic low-grade inflammation or inflammaging. Low-grade inflammation is associated with an increased risk for atherosclerosis and cardiovascular disease, and this inflammation is promoted by visceral adiposity. The authors relate to the epicardial adipose tissue, which constitutes the visceral fat depot of the heart, and which has been shown to be associated with a number of heart diseases.

The study by de Mello et al. helps to contribute further evidence in improving the healthcare of older adults. The authors aimed to determine the appropriate drug scheduling regimen for the administration of L-thyroxine in treating hypothyroidism in those older than 60 years of age. They found that thyroid-stimulating hormone (TSH) levels were similar during the follow-up period, and that there was no significant difference between morning or bedtime doses. This finding has clinical ramifications for drug scheduling in older adults.

Prostate cancer is the second leading cause of cancer death in American men, and the incidence increases with age. The two most important treatment modalities for localized prostate cancer are radical prostatectomy and intensity-modulated radiation therapy. Wu et al. conducted an important study to evaluate the differences in long-term medical resource consumption between these two forms of treatment. They found that the long-term medical resource consumption was higher in older men with high-risk localized prostate cancer undergoing intensity-modulated radiation therapy than in those undergoing radical prostatectomy.

The World Health Organization Clinical Consortium on healthy aging focused on frailty and intrinsic capacity (5). A clear emphasis was placed on functional ability, representing a combination of the intrinsic capacity of individuals, the environments they inhabit, and the interaction between them. Zhao et al. found that a higher impairment in intrinsic capacity at baseline was more likely to manifest with functional disability after a year than those with multimorbidity (three or more chronic diseases).

I believe that this Research Topic provides the researcher and clinician with interesting and varied *Insights in Geriatric Medicine* and serves as an important addition to the growing field of knowledge in Geriatric Medicine.

## Author contributions

TD: Conceptualization, Writing – original draft.
